# Customer's Perception and Preference towards Packaged Drinking Water

**DOI:** 10.1155/2020/6353928

**Published:** 2020-02-29

**Authors:** Minyahel Tilahun, Melaku Beshaw

**Affiliations:** ^1^Department of Animal Production and Technology, Wolkite University, Wolkite, Ethiopia; ^2^Department of Management, College of Business and Economics, Arba Minch University, Arba Minch, Ethiopia

## Abstract

Two hundred customers were purposively selected from two study areas (market, residence) in Addis Ababa to assess customer's behavior and perception towards packaged water. The sampling and data collection process of the study followed systematic analysis of Theory Planned Behavior. The average monthly income of respondents of this study lay between 5000 ($175) and 10000 ($350) Eth Birr. The primary customer information sources were television and radio. Residence place customers were more concerned about health as compared to market place customers. Market place customers primarily gave emphasis to the price of packaged water. Almost all (97%) customers were not sentient to packaged water standards. However, only few, 86 (43%), customers checked labeled chemical composition, of which 74 (85%) did not understand it. Customer's sex, education level, and health status showed significant relationship with choice of packaged water quality, −1.42 (*p* < 0.05); price, −2.45 (*p* < 0.01); and health status, –1.80 (*p* < 0.05) in market place and residence place, respectively. Customers were not well aware of what they were purchasing and even customer's ability to read was not related to customer's ability to understand what was written in the labels. Customers' choice of packaged drinking water has been challenged by their health status. Customers are becoming more concerned about prices while they are out of their residence place.

## 1. Introduction

Safe drinking water is one of the basic requirements for human health, development, and wellbeing [1]. Access to safe drinking water and hygienic way of living is a global concern, and the issue is especially serious in developing countries [2]. Addis Ababa is already suffering from water scarcity, which is expected to become even more significant due to rapid urbanization, increased individual water demand as incomes rise, and the impacts of climate change [3]. People are becoming more health conscious and are more careful toward drinking water.

Packaged drinking water has been taken as safe means of drinking water provision. Global packaged drinking water industry is estimated to be the most dynamic sector of all the food and beverage industry. Bottled water consumption is estimated to have reached nearly 100 billion gallons in 2017 [4]. The product has been passed through several water supply models which are already established and tested, proving their effectiveness. Given the prevailing social and technical cost needed to revitalize or put in place functional public institutions, associated technologies, and political power, it is much undoubted that the standard industrialized world model for delivery of safe drinking water technology may not be affordable in most of the developing world countries [5]. Currently in Ethiopia, people often drink packaged drinking water for different reasons such as being an alternative to tap water scarcity, contamination, and quality.

Addis Ababa, the capital city of Ethiopia and the center of the African Union, has a population of over 5 million [6]. Per capita distribution of piped water of the city is estimated to be around 40 liters/day, well below the city's goal of 110 litres/day [3]. Around 87% of drinking water source is piped water in yard or plot. The city produces 450,000 m^3^/day of piped water sourced from surface and groundwater, basically from two dams, namely, Legedadi and Gefersa dams, and from water wells [3]. The World Bank Group report in 2015 indicates that 44% of Addis Ababa populations have access to safe water supply. Nevertheless, a study conducted by Addis Ababa Institute of Technology indicates that average daily per capita consumption is below the original figure because about 37% of water produced is lost before reaching residents. Another report from Ethiopian Central Statistics Authority indicates that about 85% of the on-premises Addis Ababa's piped water is of low risk. Similarly, nationally about 42% of on-premises piped water is of low risk [3, 6]. As per the report of AAWS 2014, 15 m^3^ of water costs 0.14 $. In 2013, the Ethiopian Standards Agency developed drinking water standards to enable customers to check whether the product is in compliance with the required standards [3, 7]. However, since the introduction of packaged drinking water technology to the country through the Highland Springs brand in 1999, it is not easy to get published information about the price of packaged drinking water with its respected quality standard.

Customers think packaged drinking water tastes better and perceive it to be safer and of better quality [8]. The major challenges of the sector which have been greatly influencing consumers' attitudes are food scandals in industrialized countries and waterborne diseases in developing countries [3]. The basic means of alleviating food and beverage scandals and foodborne diseases is developing standards [9]. However, even in the presence of packaged drinking water quality standards, different scholars [10–12] indicated that heterotrophic organisms and pseudomonas species are found above the recommended level in majority of packaged drinking water available in Addis Ababa.

Consumer behavior is the study of when, why, how, and where people do or do not buy a product and it blends elements from psychology, sociology, social anthropology, and economics [13]. Even if the significance of packaged water is not doubtful, there are different constraints that customers should consider when buying packaged water. Customer's complaints about packaged water have been regularly arising mainly due to products storage and handling schemes [14]. Majority of customers do not identify points where they can get genuine information on the pros and cons of the packaged drinking water available on the market except media's advertisings. In Ethiopia there are at least four standards directly associated with water bottling-packaging and labeling, and specification for bottled drinking water and standards on plastic materials for food contact use. Ethiopian ministry of Trade gives licences after ensuring that the bottles obtain a quality performance certificate from the food, medicine, and health care administration and control authority. In 2014, Ethiopian Ministry of Trade (MoTr) gives a certificate for small number of water packaging industry brands produced in Ethiopia [15].

Although there is evidence of consumer's positive attitudes toward packaged drinking water, there remain vacant gap and frequent customers claims. These can be related to customer's brand shift. Previous studies about packaged water predominantly focus on its production, regulation, sales, consumption, criticism, and concerns. However, few researchers [14, 16, 17] have studied the relationship between consumer use of packaged drinking water and their perception level of quality and its related factors. In addition, customer's choice appropriateness in comparison to the actual condition of packaged water available in the market has not been studied well. There can be little serious doubt that health knowledge and attitude changes consequently contribute not only to individual customer behavior but also to population behavior changes over time [18]. A theoretical framework which suggests that customer's perception and attitude change toward the health and economic benefit of purchasing packaged water is a key process which should be given emphasis [19–21]. It would be unwise to take an excessively simplistic and reductionist approach toward benefiting customer's public health and securing their economic benefit. Hence, a study which can answer the question “what packaged water quality parameters should be given major emphasis while purchasing packaged water” is needed. In addition, understanding the customer's knowledge level and awareness of packaged water standards can help to secure customer's health and prevent market related abuses. The specific objectives of this study was to (i) assess customer's perception of their choice of packaged water, (ii) assess customer's level of awareness of packaged drinking water trade standards, and (iii) compare and contrast customers habits, perception level, and purpose of packaged drinking water between market and residence place.

### 1.1. Theoretical Framework

**Figure 1:**
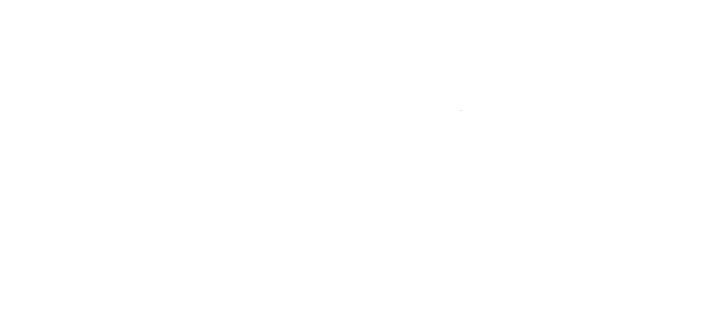
Ajzen TPB model.

Overall, the disease burden in developing countries has two main features: it occurs at much younger ages than the disease burden in developed countries, and its main channels of morbidity and mortality are infectious and parasitic diseases, which generate important public health externalities [22]. The unadulterated fact is that households in low-income countries spend a significant portion of their resources on remedial health care. In the early 2000s, bed-net coverage and point-of-use water chlorination in sub-Saharan Africa were both estimated to be fewer than 10% [23, 24]. The main reason for not acquiring preventive health products for most low-income generating households was financial constraints [25]. Indeed, demand for these products appears quite price elastic. Since 1950s evaluation of customer's behavior while purchasing healthy products was extensively performed using health behavior theories and models. This has been used to make things clear from the customer's side and understand customer's consumption behavior [26–29]. Model components should have the potential to provide a relatively comprehensive understanding of the influence of social, economic, and environmental factors on health behavior [30]. One of the well-known and acknowledged models has been the Health Belief Model (HBM). This model indicates that individual cognitive perception level about risks can be moderated by perceived benefits and barriers associated with a particular interest of the customers, which in return helps to predict customer's behavior [26–29]. The application of HBM suggests that customers purchasing decision can be influenced by their level of perception of contamination causes and availability of understandable information, which are used as central indicators for model evaluation. Although a number of documents have been supporting the influence of HBM variables on health behavior, ambiguity still exists concerning identifying most significant variables and their interaction within the model [31, 32]. Hence, the HBM is not in itself clearly or adequately specified, and the available evidence indicates that in practice its application appears to be inadequate for such purposes [33]. For many households in developing countries investing cash which is not comparable with monthly income generated demand for information about the product they buy. Preventive health tools such as drinking safe source water remain low unless they can have credit access. Money invested in treating disease cannot be invested in preventive health. The other theory, which is Theory Planned Behavior (TPB), touches areas of education and information, social learning, financial markets, credit constraints, preference, healthy behavior, and policy implication (Figure 1). Systematic analyses which incorporate full range of components might cast light on the impact of social and other factors on inequalities in health and the reasons why individuals and groups may not take up health improvement or protection opportunities [33]. The theory in some respects is a refining and taking forward approach which is embodied in the HBM. This model design and dissemination follow Bandura's [34] on self-efficacy and the publication of his Social Cognitive Theory [35, 36].

TPB is superior to that of the HBM, and also the additional constructs contained in the TPB allow it to predict a greater percentage of overall behavioral variances [33]. In general, all models appeared to have the potential to increase the sustained adoption of preventive behavior. However, the success of these demand-side strategies is contingent on the supply side being adequate: on health services and products being available, with delivery and/or enforcement institutions that are effective. As indicated in Ajzen TPB model, among the basic components of the model, the significance of customer's perceived behavior change in creating an intention which can critically understand what to buy and use is strongly correlated with customer's perception level, customer's social norm, and other additional components (Figure 1). In addition, different norms which are acquired from the society should be given great emphasis while trying to assess customer's perception and attitude level toward some goods which they want to buy. Customers perception difference can elucidate the specific gap and gearing variable which need due emphasis and it also eases interpretation of results and comparison of findings across studies undertaken in differently identified problematic area. This study used components of TPB, i.e., customer's perceived behavioral and social norms, as a reference framework to attain the above objectives.

## 2. Materials and Methods

### 2.1. Study Area

Addis Ababa is the capital city of Ethiopia which is located at the center of the country between 8°55′ and 9°05′N and 38°40′ and 38°50′E. Its altitude ranges from 2000 to 2800 m.a.s.l [37]. The city has a total population of 3,384,569 according to [38]. The city hosts two major rain seasons; i.e., the short rain season occurs between March and May, while the main rain season occurs between June and September. The dry season occurs during the months of October and February [39].

### 2.2. Data Type and Data Collection Method

This study used both primary and secondary data source. Primary data was collected using semistructured questionnaire using formal survey. In addition, personal observation on quality of the product kept for seal, group of customers (age, sex) who frequently purchase packaged water, frequently asked questions about the products, customers understanding level of the label, and questions related to price were given major emphasis. The questionnaire included questions based on attitude, subjective norms (SN), perceived behavioral control (PBC), and behavioral intention. Questions based on demographic and sociodemographic data also used different secondary information sources such as bulletin and flayers which described the drinking water access and distribution around Addis Ababa.

### 2.3. Research and Sampling Design

Descriptive research design which followed purposive sampling technique was undertaken between May and September, 2018. Customers were selected based on their experience and frequency of purchasing packaged drinking water per week. Customers who had more than two years of packaged drinking water consumption experience and customers who bought a minimum of three litres of packaged water per week were considered for this study. In addition, this study considered two common consumers groups from two major areas of a city, i.e., markets place and residence place. Two study sites which represent the market place, i.e., *Merkato* and *Shola* markets, and *CMC* and *Sarbet* which represent the residence place were selected based on their intensity of hosting different packaged drinking water customers and sellers. In addition, the selected sites hosted both localized customers to a specific place and frequently traveling customers whose destinations were unknown. A total of 200 customers/respondents, i.e., 100 from each study area, were involved in this study. In addition to the formal survey and personal observation, triangulation of the collected data was done by interviewing 50 retailers (25 from each study area) using similar questionnaire.

### 2.4. Data Analysis

The collected data were debugged and scrutinized before starting the analysis. SPSS version 20 was used to calculate descriptive statistics and index of different variables to assess the purchasing behavior of packaged drinking water customers. In addition, multinomial logistic regression model was calculated to assess customer preference while purchasing packaged drinking water.

(1) $$\begin{array}{c} nij=\alpha j+xi\beta j, \end{array}$$

where *αj* is a constant and *βj* is a vector of regression coefficients, for *j* = 1, 2,…, *J *−* *1. Index was computed to provide the overall rank of customer's source of media and preference while purchasing packaged water using the following formula.

(2) $$\begin{array}{c} \text{Index}=\frac{R_{n}*C_{1}+R_{n}-1*C_{2}+\cdots +R_{1}*C_{n}}{\Sigma R_{n}*C_{1}+R_{n}-1*C_{2}+\cdots +R_{1}*C_{n}}, \end{array}$$

where *R*_*n*_ is the value given for the least ranked level (for example, if the least rank is 5th, then *R*_*n*_ = 5, R_*n*−1_ = 4, and…, *R*_1_ = 1); *C*_*n*_ is the count of the least ranked level (in the above example, the count of the 5th rank = *C*_*n*_, and the count of the 1st rank = *C*_1_).

## 3. Results

### 3.1. Household Characteristics

Table 1 describes the descriptive statistics of the respondents from the study areas. The average age of respondents purchasing packaged drinking water fell between 34 and 35 years. In the study, 106 (53%) of female and 94 (47%) of male groups participated. Residence place respondents were represented by slight higher (59%) female group as compared to market place (47%). Majority (78%) of the respondents participating in the study had an education level of above secondary school, and higher education level proportion (35%) was observed in residence place respondents.

About 76% of the respondents involved in the study had private means of occupation. The average family size of the respondents in the study was four. Around 60% of the respondents of the study had an average monthly income of below 10000 ETB. Only 73 (36.5%) of the respondents experienced health issues (illness and pregnancy) which demanded consumption of packaged water, of which the majority 39 (53%) were due to neonate feeding and pregnancy.

### 3.2. Customer's Awareness of Packaged Water

Table 2 describes the sources of media used for creating awareness. Television and radio 675 (0.34) were the major media used for creating awareness followed by colleagues, 572 (0.29), and newspapers and magazines, 450 (0.23). Even though the access of Internet showed a great advancement, the result indicated that use of window display to create awareness is still very minor 271 (0.14).

### 3.3. Customer's Preference and Reasons for Purchasing Packaged Water

Customer preference and reasons for purchasing packaged drinking water are presented in Table 3. Customers purchased packaged water for different reasons; among these, the basic ones were domestic travel, water scarcity, contamination, and restaurants and hotels service. The primary reason for purchasing packaged water for market place respondents was restaurants and hotels service, 603 (0.33), followed by traveling alone (long journey), contamination of water, and scarcity of water and domestic travel, respectively. However, contamination of tap water was the primary reason for purchasing packaged drinking water for residence place respondents. Scarcity of water, domestic travel, traveling alone, and restaurants and hotel service were ranked second, third, and fourth, respectively. Price, 378 (0.38), of packaged water was the primary factor behind market place customer's preference followed by test and packaging materials of the packaged water. On the contrary, quality of the packaged water, 282 (0.28), was the base for resident place respondents for choosing their product followed by price and packaging material.

### 3.4. Customer's Satisfaction with Packaged Drinking Water

Customer's satisfaction with packaged water is presented in Table 4. The average amount of packaged drinking water purchased by respondents was 7.7 litres. The study indicated that much higher amount (11.29 ± 15.05) of packaged drinking water was purchased per week by residence place customers than market place customers (4.1 ± 3.04). The majority (26.5%) of customers indicated that their satisfaction level was good. The satisfaction level of sixty-eight percent of residence place respondents ranged between the levels of good and very good, which was higher than this of market place respondents, which was 33%. The average amount of money spent by market place customers to purchase packaged water was 401 ETB per week, which is so much lower than the money spent by residence place customers (i.e., 1129 ETB per week).

### 3.5. Customer's Complaints against Packaged Water

Table 5 presents customer's complaints against packaged water. The present study revealed that a total of 113 (61.5%) customers disclosed their complaints against packaged drinking water. Among them, the majority (62%) of market place customer's complaints were related to price. On the other hand, packaged water quality (40%) was the major complaint for residence place customers. Generally, price (43%) was the primary area of complaint followed by quality and test. The majority of customers (79%) thought that producers are responsible for the forwarded complaints.

In general, almost half of the respondents shifted their packaged water brand for different reasons. Of them, the majority (77%) were market place customers. 76% of residence place respondents were loyal to frequently used brand as compared to market place customers (23%). The major reasons for shifting brands in the market places were new product development (53.25%) and new production technology (22.08%). On the contrary, availability of the product in the market (41.67%) was the primary reason followed by new product development (16.67%) for residence place customers.

### 3.6. Customer's Knowledge of Packaged Water Standards

Table 6 describes customer's and retailer's awareness of packaged water trade standards and quality. The study indicated that only few, 8 (3.2%), of the customers and retailers were capable of describing packaged drinking water standards. Retailers primarily gave emphasis to packaged water price (50%) as compared to other informative variables. However, majority (38%) of the customers observed chemical composition which was presented on the packaged drinking water. Among these, 70% of residence place customers primarily gave emphasis to chemical composition (56%) and manufacturing date (14%). Among the customers who observed the presented chemical composition, only 13 (15%) understood what was presented on the labels. The primary reason for not understanding the presented chemical composition on the label was basically its presentation in scientific writing, 31 (12.4%). Around 80% of the respondents reported that they did not find the type of packaged drinking water they requested.

### 3.7. Customer's Primary Emphasis while Purchasing Packaged Water

Table 7 describes customer's characteristics in relation to their preference while purchasing packaged drinking water. Identifying the basic factors which have significant effect on customers can show producers what the consumers are seeking in the market. Basic factors such as status of residence, health status, and average income of the regular customers were the primary causes for shifting from brand to brand. The study indicated that price had a negative relationship with both study areas, i.e., market place (−2.45) and residence place (−2.61), with customers educational level being at *p* = 0.05 and *p* = 0.01, respectively. Other demographic variables did not show a significant relationship with customer's preference while buying packaged water. Being a female customer from the market place had a negative significant relationship with packaged water quality with a coefficient of −1.42. Packaged water quality had also a negative relationship with health status of residence place customers with a coefficient of −1.80.

## 4. Discussion

Drinking water has no taste and it is challenging to consumers to analyze the quality of drinking water using sensory organs [40]. Both HBM and TPB models indicate that value placed by customers and estimates of likelihood of a given action will achieve the demanded goal. Conceptualizing the context of the models can grant a minimized risk of disease occurrence and supply of proper quality of packaged drinking water [33]. These models depend on SN and PBC which can determine the occurrence of basic waterborne diseases which are prevalent in the developing world. Consumers' understanding of the presented label and the different information presented through media is associated with level of perception and level of education. In developing countries where literacy level is low, this information can only benefit few of packaged drinking water customers who are obliged to consume restricted amount of trace minerals. Similar to customer's behavior model, many decisions of purchasing different packaged drinking water brands have not coincided with proper awareness of the consumers. In contrast to [41], majority (43%) of respondents who purchased packaged drinking water are able to understand contents of the label presented on the bottle. Customer's understanding ability might be directly related to their level of education which enables them to read the type of language used to present the information on the label. The finding of this study is in contrast with [42] conducted in Sir Lanka which reported that customer's educational level has no significant relationship with customer's behavior. Moreover, higher living standards might enable customers to customize the available information and easily bring packaged drinking water home more frequently [26]. In the developing countries, the relationship between customer's level of income and trends of purchasing packaged drinking water indicates that many developing countries are good markets for packaged drinking water [4]. However, delivery of safe drinking water technologies may not be easily affordable by low-income countries [5]. Besides, many factors including demographic variables, quality of water sources, and trust in tap water companies also seem to influence public behavior [27].

Some customers might pay attention to factors such as health, physiology, and ecology that inhibit them from consuming the average amount of trace minerals. Packaged drinking water's basic quality is working on trace minerals which are necessary for healthy individuals on a daily basis. Different organizations report the standardized amount of trace minerals for a litre of packaged water [7, 28]. The purchasing stimuli of packaged drinking water customers are in line with the study in [29] which indicates that the winding variable to purchase packaged drinking water is economic, political, and cultural circumstances of a society. In major product markets, media have been helping in the improvement of customer's awareness, which might also vary with the geographical location and demographic and coverage level of media [41]. Respondents from the market place mostly give emphasis to packaged drinking water price, and this result is in line with that in [16] which indicated that consumers were charged unfair price of packaged water. Ecura et al. [26] reported that domestic drinking water of developing countries is treated with chlorine and maintains a certain concentration of residual chlorine to disinfect potential bacterial contamination. As to [27], health, taste, water quality, lifestyle, environment, and perceived alternatives are all correlated with packaged drinking water consumption. Personal observation of the researchers showed that the last product sold to customers is commonly covered with dirt and dust, and, in majority of the retailing shops, packaged drinking water has been stored in front of the shops and open to direct sunlight. Matiwos [16] reported that dust and stain on packaged water during storage degrades quality of packaged water. Similarly, Amogne [14] reported that problems related to packaged drinking water quality are basically arising due to storage and handling schemes of the products. In line with this study finding, the most preferred Ghanaian bottle water brands received the least rating for quality complaints [43]. The studies [14, 44] are in contrast to this finding in which sellers are more responsible than producers for the degradation of water quality rising due to package and storage. The observed difference between those studies and this study might be attributed to the type of methodology applied and the type of quality of the water to be assessed. Furthermore, due to customer's levels of understanding and preference level toward packaged water quality in the two differently presented study areas, i.e., market places and residence places of Addis Ababa, the reasons for their choice of drinking packaged water might ultimately differ from the expected output of the study. Throughout the world, the number of packaged drinking water customers is increasing from year to year [4]. The average amount of packaged water (7.7 litres) purchased by customers of this study is a little bit higher than that in [16] which was 4-5 litres of packaged water per week. The observed difference is attributed to the methodology used for this study, i.e., selecting respondents who have the experience of purchasing a minimum of three litres of packaged drinking water per week. The sales volume of packaged water in developed countries like Singapore hit $134 million in 2015 [26].

An estimate coefficient of 2.45 (*p* < 0.01) for customer's level of education is attributed to an increase in customer's demand for low price packaged drinking water. This suggests that one-step increase in customer's educational level might increase their concern regarding packaged water price while purchasing. Female customers are more careful in purchasing quality packaged drinking water as compared to male consumers. This might be the reason why female customers become more loyal to a brand than male customers. Most customers with health issues are more likely to choose their packaged water in terms of quality as compared to other customers by a rate of -1.8 (*p* < 0.05). However, residence place customers give more emphasis to quality of the packaged water than other variables. The finding of this study is in contrast with that in [45] which reported that color, graphic design, size, and shape of packaging of packaged drinking water significantly influence consumers' decision of purchasing packaged drinking water. The result of [45] is in contrast with this study; it indicated that customers are health conscious while aiming to purchase packaged water. The negative relationship between the health status of residence place customers and the quality of packaged water might be attributed to the fact that customers become more concerned about the quality of the packaged water when they are in bad health condition, which makes them rely only on packaged water rather than using other water sources like tap water.

Consumers from both study areas often drink packaged drinking water as an alternative to tap water. The major reasons for purchasing packaged water were particularly related to accessibility, health issue, and contamination or cleanness. The primary reason for residence place customers is their health concern, whereas customers from market place primarily purchase packaged water as a substitute for tap water while they are using restaurants and hotels and while they are traveling. Less than half of packaged drinking water customers check the chemical composition on the label while purchasing. However, majority of them do not understand what is presented in the labels. Customer's sex, literacy level, and health status have significant relationship with price and quality of packaged drinking water while purchasing.

Customer's level of knowledge about a product can help them to compare available products and purchase the right product. It is significantly related to customer' literacy level and understanding level of foreign language and chemical composition. Awareness creation programs concerning packaged water standards by considering customer's age, physiological level, and literacy level should be prepared and delivered to improve customer's knowledge of packaged water quality and standards. Furthermore, labels should be easily understandable and informative. In addition, further studies should focus on the possibilities of verifying the origin, physical microbiological quality, and authenticity of packaged drinking water. Identifying the consequences of packaged water production which does not follow the available standards, its impact on people health, and the different causes of packaged water contamination should be given due emphasis.

## Figures and Tables

**Table 1:** Descriptive statistics of respondents from the study areas.

Descriptive variable	Study area		Total (*N* = 200)
	Market (*n* = 100)	Residence place (*n* = 100)	
Age (mean ± SD)	34.86 ± 10.8	34.73 ± 11.33	34.8 ± 11.04

Sex			
Male	53	41	94 (47)
Female	47	59	106 (53)

Educational level			
Illiterate	5	2	7 (3.5)
Elementary	21	15	36 (18)
Secondary school	47	48	95 (47.5)
Above college diploma	27	35	62 (31)

Type of occupation			
Private	72	83	155 (77.5%)
Public	28	17	45 (22.5%)

Family size (mean ± SD)	4.06 ± 2.24	4.78 ± 1.85	4.42 ± 2.05

Nature of housing			
Private	34	71	105 (52.5%)
Renting	66	29	95 (47.5%)

Monthly income/ETB			
Below 5,000	30	25	55 (27.5%)
5,000–10,000	45	26	71 (35.5%)
10,000–15,000	12	37	49 (24.5%)
Above 15,000	3	12	15 (7.5%)

Health issues (yes)	31	42	73 (36.5%)

Type of health problem			
Chronic disease	24	10	34 (46.6)
Pregnancy and serving neonate	7	32	39 (53.4)

Note that “ETB” represents the local currency of Ethiopia. At the time of undertaking this study 1 ETB exchange rate was close to 0.28$. The row “health issues” presented only “yes” choices which helped in further analysis.

**Table 2:** The sources of media used to create awareness of packaged water.

Media	Study area				Total (*N* = 200)	
	Market (*n* = 100)		Residence (*n* = 100)			
	*N* (index)	Rank	*N* (index)	Rank	*N* (index)	Rank
Newspapers and magazines	215 (0.22)	3	235 (0.24)	3	450 (0.23)	3
Television and radio	364 (0.36)	1	312 (0.32)	1	675 (0.34)	1
Window display	120 (0.12)	4	151 (0.16)	4	271 (0.14)	4
Colleagues	301 (0.30)	2	271 (0.28)	2	572 (0.29)	2

**Table 3:** The rank of customer's reasons and preference^*∗*^ of buying packaged water.

Reason for purchasing and preference^*∗*^	Market (*n* = 100)		Residence place (*n* = 100)		Total (*N* = 200)	
	*N* (index)	Rank	*N* (index)	Rank	*N* (index)	Rank
Traveling alone	359 (0.20)	2	266 (0.19)	4	625 (0.21)	2
Travel and domestic use	263 (0.14)	5	272 (0.19)	3	535 (0.18)	5
Scarcity of water	285 (0.16)	4	292 (0.21)	2	577 (0.19)	4
Contamination in tap water	315 (0.17)	3	302 (0.21)	1	617 (0.20)	3
Restaurant and hotel service	603 (0.33)	1	264 (0.19)	5	667 (0.22)	1
Packaging material^*∗*^	192 (0.20)	3	247 (0.25)	3	439 (0.22)	3
Quality^*∗*^	183 (0.18)	4	282 (0.28)	1	465 (0.23)	2
Price^*∗*^	378 (0.38)	1	264 (0.26)	2	642 (0.32)	1
Test^*∗*^	234 (0.24)	2	204 (0.20)	4	438 (0.22)	4

**Table 4:** The amount of packaged water purchased per week and customers attitude toward their level of satisfaction (freq (%)).

Consumers level of satisfaction	Study area		Total (*N* = 200)
	Market (*n* = 100)	Residence place (*n* = 100)	
Packaged water quantity consumed			
Liter/wk.	4.1 ± 3.04	11.29 ± 15.05	7.7^*∗*^ ± 11.41
ETB/wk.	401	1129	765^*∗*^

Level of customer satisfaction			
Poor	33	12	45 (22.5)
Satisfactory	13	09	21 (10.5)
Good	19	34	53 (26.5)
Very good	14	34	48 (24)
Excellent	21	11	32 (16)

Note that the study included only respondents who purchase 3 litres of packaged water; ^*∗*^value; frequency and percentage values in both study areas are the same and are presented by single value.

**Table 5:** Customer's complaints against packaged water.

Complaint	Study area (*n* (%))		Total (*N* (%))
	Market place	Residence place	
Presence of complaint (yes)	63	50	113 (61.5)

Area of complaint			
Packing	1 (1.6)	10 (20)	11 (10.8)
Quality	14 (22.2)	20 (40)	34 (31.1)
Price	39 (61.9)	12 (24)	51 (42.9)
Taste	9 (14.3)	8 (16)	17 (15.15)

Who is responsible for your complaint?			
Producer	46 (73)	41 (82)	87 (79)
Seller	8 (12.7)	5 (10)	13 (10.5)
Both	9 (14.3)	4 (8)	13 (10.5)

Brand loyalty (yes)	23	76	99 (49.8)
Shifting (yes)	77	24	101 (50.5)

Major reasons for shifting brands			
Availability	5 (6.5)	10 (41.67)	15 (14.85)
Taste	8 (10.39)	2 (8.33)	10 (10.1)
Package size	6 (7.8)	6 (25)	12 (11.88)
New product	41 (53.25)	4 (16.67)	45 (44.55)
New production technology	17 (22.08)	2 (8.33)	19 (18.81)

Note that the rows “presence of complaint,” “brand loyalty,” and “shifting” presented only “Yes” choice, which helped in further analysis.

**Table 6:** Customer's and retailer's knowledge of packaged water trade standards and quality (*n* (%); *N* (%)).

Knowledge of standards and quality	Customers (*N* = 200)			Retailers (*n* = 50)	Total (*N* = 250)
	Market place	Residence place	Total		
Describing trade standards	1 (1)	2 (2)	3 (3)	5 (10)	8 (3.2)

What part of the label do you observe before buying?					
None	24	2	26	8 (16)	34 (13.6)
Manufacture date	12	14	26	1 (2)	27 (10.8)
The chemical composition	30	56	86	5 (10)	91 (36.4)
Price	31	11	42	25 (50)	67 (26.8)
All	3	17	20	11 (22)	31 (12.4)

Do you understand chem. composition? (*n* = 86; 5)					
Yes	4 (13.33)	9 (16)	13 (15.2)	1 (20)	14 (15.4)
No	26 (86.67)	47 (84)	73 (84.8)	4 (80)	77 (84.6)

Reasons for low level of understanding					
Illiteracy	4	1	5	—	5 (2)
Scientific writing	22	5	27	4 (80)	31 (12.4)
Foreign language	14	4	18	—	18 (7.2)
All	17	6	23	—	23 (9.2)

Did you find what you required?					
Yes	10	32	42 (21)	18 (36)	60 (24)
No	90	68	158 (79)	32 (64)	190 (76)

Note that the row “do you understand chem. composition?” was associated with the response collected from the row “what part of the label do you observe before buying?”.

**Table 7:** Customer's preference in relation to their demographic characteristics.

Customers primary emphasis	Age			Sex			Education level			Nature of the house (rented)			Income/month			Source of income			Health status		
	B (SE)	Wald	Sig.	B (SE)	Wald	Sig.	B (SE)	Wald	Sig.	B (SE)	Wald	Sig.	B (SE)	Wald	Sig.	B (SE)	Wald	Sig.	B (SE)	Wald	Sig.
Price	−023 (0.39)	0.33	0.57	−0.13 (0.93)	0.02	0.89	−2.45 (0.84)	8.51	**0.004** ^*∗∗*^	0.59 (0.91)	0.42	0.52	−0.19 (0.56)	0.12	0.73	0.15 (0.30)	0.25	0.62	1.32 (1.21)	1.18	0.28
Taste	0.07 (0.51)	0.02	0.89	−0.64 (1.2)	0.29	0.59	−0.71 (1.04)	0.47	0.49	1.59 (1.28)	1.55	0.21	0.53 (0.75)	0.49	0.48	0.14 (0.39)	0.13	0.72	−0.76 (1.25)	0.37	0.54
Accessibility	−0.002	0.00	0.10	0.64 (0.92)	0.49	0.48	−0.71 (0.85)	0.71	0.40	0.75 (0.80)	0.87	0.35	−0.05 (0.48)	0.01	0.92	0.12 (0.27)	0.19	0.66	0.16 (0.94)	0.03	0.86
Quality	−0.20 (0.29)	0.46	0.50	−1.42 (0.72)	3.85	**0.050** ^*∗*^	0.41 (0.84)	0.24	0.63	−0.02 (0.67)	0.00	0.97	0.45 (0.41)	1.19	0.28	0.21 (0.23)	0.85	0.36	−0.64 (0.80)	0.64	0.43
Price^X^	−0.21 (0.42)	0.26	0.61	−0.86 (0.97)	0.80	0.37	−2.61 (0.96)	7.43	**0.01** ^*∗∗*^	0.45 (0.92)	0.24	0.62	0.17 (0.59)	0.09	0.77	0.04 (0.31)	0.01	0.91	0.76 (0.97)	0.62	0.43
Taste^X^	0.26 (0.62)	0.18	0.67	−0.51 (1.25)	0.17	0.68	−0.32 (1.33)	0.06	0.81	2.22 (1.52)	2.14	0.14	0.37 (0.98)	0.22	0.64	−0.07 (0.43)	0.02	0.88	1.92 (1.39)	1.90	0.17
Accessibility^X^	0.17 (0.37)	0.20	0.66	0.62 (0.95)	0.42	0.52	−1.44 (0.97)	2.21	0.14	0.91 (0.86)	1.13	0.29	−0.13 (0.53)	0.06	0.80	0.07 (0.29)	0.053	0.82	−0.44 (0.89)	0.24	0.63
Quality^X^	−0.29 (0.31)	0.90	0.34	−0.54 (0.74)	0.54	0.46	−1.41 (0.90)	2.50	0.113	0.30 (0.70)	0.18	0.67	0.34 (0.45)	0.59	0.44	0.11 (0.24)	0.188	0.66	−1.80 (0.81)	4.93	**0.02** ^*∗*^
Pseudo-*R*^2^	Residence place	Market place																			
Cox and Snell	0.57	0.461																			
Nagelkerke	0.594	0.491																			
McFadden	0.262	0.227																			

^*∗*^ *p* value of 0.05; ^*∗∗*^*p* value of less than or equal to 0.01; ^x^residence place.

## Data Availability

Data cannot be publicly shared for the sake of security.

## References

[1] Khallaf E. A., Galal M., El-Sbbagh S., Nabet N. M. (2014). A study of the physico-chemical characteristics of raw, filtered, and treated water at a water treatment plant in Shebin El-Kom, Egypt. *Egyptian Journal of Aquatic Biology and Fisheries*.

[2] Amenu D. (2013). Drinking water quality. *World Journal of Arts, Commerce and Sciences*.

[3] The World Bank Group (2015). *Enhancing Urban Resilience. Global Practice on Social, Urban, Rural and Resilience*.

[4] John G. R. (2017). Statistics. https://www.bottledwater.org/.

[5] Lawrence Y. K., Senyo A., Kwamena M. N. (2015). Challenges and prospects confronting commercial water production and distribution industry: a case study of the cape coast metropolis. *International Journal of Management Sciences*.

[6] Central Statistical Agency (CSA) (2017). Drinking water quality in Ethiopia; results from the 2016 Ethiopia socioeconomic survey.

[7] Compulsory Ethiopian Standard (CES) (2013). *Drinking Water Specification*.

[8] Miguel F. D. (2006). Bottled water versus tap water: understanding consumers’ preferences. *Journal of Water and Health*.

[9] Dinka M. O. (2019). Safe drinking water: concepts, benefits, principles and standards. *Water Challenges of an Urbanizing World*.

[10] Bedada T. L., Dera F. A., Edicho R. M. (2018). Mycological and bacteriological quality and safety of bottled water in Ethiopia. *The Open Microbiology Journal*.

[11] Tafere W., Abera F., Beyene Y., Legesse T. (2016). Microbiological quality and safety of bottled water brands sold in Ethiopia. *The Ethiopian Journal of Health Development (EJHD)*.

[12] Biadglegne F., Tessema B., Abera B. (2009). Physicochemical and bacteriological quality of bottled drinking water in three sites of Amhara Regional State, Ethiopia. *Ethiopian Medical Journal*.

[13] Walter R. N. (1974). *Organizational Behaviour-Human Behaviour at Work*.

[14] Amogne W. T. (2016). Labelling practices of water bottling firms and its public health perspective in Ethiopia. *Ethiopian Journal of Health Development*.

[15] Ministry of Trade (MoTr) (2014). *Annual Report on Water Provision and Conservation*.

[16] Matiwos E. (2014). Trends in bottled water use survey in Addis Ababa: implication on reverse logistics of bottled water manufacturing in Ethiopia. *International Journal of Science and Research (IJSR)*.

[17] Dawit K. M. (2015). The effect of distribution systems on household drinking water quality in Addis Ababa, Ethiopia, and Christchurch, New Zealand.

[18] Sajjadi A. S., Alipour V., Matlabi M., Biglari H. (2016). Consumer perception and preference of drinking water sources. *Electronic Physician*.

[19] Ahmed D. M., Kranthi N. (2018). Conceptual framework for water poverty. *International Journal of Applied Engineering Research*.

[20] Dreibelbis R., Winch P. J., Leontsini E. (2013). The integrated behavioural model for water, sanitation, and hygiene: a systematic review of behavioural models and a framework for designing and evaluating behaviour change interventions in infrastructure-restricted settings. *BMC Public Health*.

[21] United Nations Department of Economic and Social Affairs (2015). *Development Goals: Synthesis of Knowledge and Recommendations for Effective Framing, Monitoring, and Capacity Development*.

[22] Florencia D., Esther D., Pascaline D., William P., Vincent P. (2012). Happiness on tap: piped water adoption in urban Morocco. *American Economic Journal, Economic Policy*.

[23] Miller J. M., Korenromp E. L., Steketee B. L., Richard R. (2007). Estimating the number of insecticide-treated nets required by African households to reach continent-wide malaria coverage targets. *JAMA*.

[24] Lauren J. S., Thea K. F., Michael D. (2007). Point-of-use water treatment and use among mothers in Malawi. *Emerging Infectious Diseases*.

[25] Guerrant R. L., Kosek M., Lima A. A., Lorntz B., Guyatt H. L. (2002). Updating the DALYs for diarrhoeal disease. *Trends in Parasitology*.

[26] Ecura J., Okot-Okumu J., Okurut T. O. (2011). Monitoring residual chlorine decay and coliform contamination in water distribution network of Kampala, Uganda. *Journal of Applied Sciences and Environmental Management*.

[27] Lim L. (2017). Singapore’s $134 m bottled water addiction. http://www.channelnewsasia.com/news/singapore/singapore-s-s-134m-bottled-water-addiction/3364034.

[28] WHO (2011). *Guidelines for drinking-water quality*.

[29] Durga M. (2010). *Consumers Buying Behaviour of Bottled Water Suriname. A Study on the Relationship between Demographic and Psychological Factors and Bottled Buying Behaviour*.

[30] Crepaz N., Marks G. (2002). Towards an understanding of sexual risk behavior in people living with HIV: a review of social, psychological, and medical findings. *AIDS*.

[31] Adams J., White M. (2003). Are activity promotion interventions based on the transtheoretical model effective? A critical review. *British Journal of Sports Medicine*.

[32] Strecher V. J., Champion V. L., Rosenstock I. M., Gochman D. S. (1997). The health belief model and health behaviour,. *Handbook of Health Behavior Research I: Personal and Social Determinants*.

[33] David T., Michael B., Natasha C. (2006). *A Review of the Use of the Health Belief Model (HBM), the Theory of Reasoned Action (TRA), the Theory of Planned Behaviour (TPB) and the Trans-theoretical Model (TTM) to Study and Predict Health Related Behaviour Change*.

[34] Bandura A., Vasta R. (1989). Social cognitive theory,. *Annals of Child Development*.

[35] Ajzen I., Kuhl J., Beckman J. (1985). From intentions to action: a theory of planned behaviour,. *Action-Control: From Cognition to Behaviour*.

[36] Ajzen I. (1988). *Attitudes, Personality, and Behaviour*.

[37] Regassa N., Sundaraa R. D., Seboka B. B. (2011). Challenges and opportunities in municipal solid waste management: the case of Addis Ababa city, Central Ethiopia. *Journal of Human Ecology*.

[38] CSA (2007). *Population and Housing Census of Ethiopia*.

[39] Hailu G. (2011). *Assessment of the Status of Nitrate Pollution in Selected Water Sources in Addis Ababa*.

[40] Eric T., Christine U., Sylvie C., Pascal S. (2010). Consumer perception and preference of bottled and tap water. *Journal of Sensory Studies*.

[41] Seifu A. T., Amy S. C., Manyahlshal A. (2012). *Water Supply and Sanitation in Amhara Region. Learning and Communication Research Report*.

[42] Delina P. JE., Dasinaa S. (2016). Consumers perception and factors influencing adapting of Bottled water consumption in Batticaloa district, Sri Lanka. *International Journal of Interdisciplinary Research Methods*.

[43] Osei EK. (2016). Assessing the effect of branding on the performance of bottled mineral water producing companies in Ghana.

[44] Yalew M. (2014). Influence of bottled water packaging attributes on consumers purchase decision. Case Study in Addis Ababa. *Journal of Basic and Applied Sciences*.

[45] Ferrier C. (2009). Bottled water. A social phenomenon. *A Journal of the Human Environment*.

